# The Clinicopathological Characteristics and Surgical Treatment of Gastrointestinal Neuroendocrine Neoplasm—A 10-Year Single-Center Experience

**DOI:** 10.3390/jcm13164892

**Published:** 2024-08-19

**Authors:** Michał Serafin, Beata Jabłońska, Emila Senderek, Karolina Majewska, Sławomir Mrowiec

**Affiliations:** 1 Student Scientific Society, Department of Digestive Tract Surgery, Faculty of Medical Sciences in Katowice, Medical University of Silesia, 14 Medyków Street, 40-752 Katowice, Poland; michal.j.serafin@gmail.com (M.S.); em.senderek@gmail.com (E.S.); 2 Department of Digestive Tract Surgery, Faculty of Medical Sciences in Katowice, Medical University of Silesia, 14 Medyków Street, 40-752 Katowice, Poland; majewskakarolina1008@gmail.com (K.M.); mrowasm@poczta.onet.pl (S.M.)

**Keywords:** gastrointestinal tract, neuroendocrine tumors, surgical treatment

## Abstract

**Background:** Gastrointestinal neuroendocrine neoplasms (GI-NENs) represent a diverse group of tumors, with surgical resection being the gold standard for treatment. **Materials and Methods:** A retrospective analysis was conducted on 63 patients (32 women, 31 men) who underwent surgery for GI-NENs at the Department of Digestive Tract Surgery from January 2013 to June 2023. Tumors were classified by stage (localized, regionally advanced, metastatic). **Results:** Clinical symptoms were reported by 42 (66.7%) patients, with abdominal pain being the most common symptom, affecting 28 (44.4%) patients. The majority of tumors (44, 69.8%) originated in the midgut. The most frequently performed surgery was right hemicolectomy, carried out on 33 (52.4%) patients. Radical tumor resection was performed in 35 (55.6%) patients. Postoperative complications occurred in 12 (19%) patients, with male gender identified as an independent predictive factor for complications (*p* = 0.04). Non-functioning tumors were more common (33, 52.4%), and most tumors were classified as grade 1 histopathologically (49, 77.8%). Distant metastases were present in 29 (46%) patients. The overall two-year survival rate was 94.9%, with a five-year survival rate also estimated at 94.9%. **Conclusions:** GI-NENs are often diagnosed at advanced stages, frequently with distant or lymph node metastases, and predominantly arise in the midgut. Despite low postoperative morbidity and mortality, male gender may be a predictor of postoperative complications. Overall, the prognosis for GI-NENs is favorable, reflected in high overall survival rates.

## 1. Introduction

Gastrointestinal neuroendocrine tumors (GI-NENs) are a diverse group of neoplasms that arise from well-differentiated secretory cells of the neuroendocrine system or their precursors (stem cells) [1,2]. These tumors are categorized based on their embryological origin into three groups: those originating from the foregut (e.g., thymus, esophagus, lung, stomach, duodenum, pancreas), midgut (e.g., appendix, ileum, cecum, ascending colon), and hindgut (e.g., distal large bowel, rectum) [3].

Initially considered rare since their discovery in 1867, the incidence of NENs has increased significantly, from 1.09 per 100,000 in 1973 to 6.98 per 100,000 in 2012 [4]. This rise is attributed to advancements in diagnostics and healthcare. Currently, NENs represent 0.5% of all malignant neoplasms and 2% of malignant GI tumors [5]. Geographic and demographic variations in the incidence of GI-NENs have been observed, with the highest rates reported in North America and Europe, where small intestinal and colonic NENs are predominant. Conversely, in Asia, rectal neuroendocrine neoplasms are the most prevalent [4,6]. The incidence of GI-NENs increases with age, peaking around the sixth decade of life [6].

Clinically, GI-NENs can be asymptomatic or present with various symptoms depending on their location and hormonal activity [7]. Functioning GI-NENs secrete hormones and biogenic amines, leading to distinct syndromes such as carcinoid syndrome, Zollinger–Ellison syndrome, and endogenous hyperinsulinemia hypoglycemia. Carcinoid syndrome, associated with serotonin-secreting tumors, is characterized by flushing, diarrhea, and abdominal cramps. Zollinger-Ellison syndrome, caused by gastrin-secreting tumors, involves severe peptic ulcers and abdominal pain due to excessive gastric acid production. Endogenous hyperinsulinemia hypoglycemia caused by insulinoma leads to hypoglycemia with symptoms such as confusion and sweating [7,8,9,10,11]. Additionally, other neuroendocrine tumors syndromes, such as VIPoma, are highlighted in the ENETS 2023 guidance [12]. Non-functioning GI-NENs typically present with symptoms related to tumor mass or metastasis, such as abdominal pain, bowel obstruction, or jaundice [7,8,9,10,11].

Currently, there is no universally accepted biomarker for GI-NENs. Chromogranin A (CgA) is one of the most commonly used biomarkers, but its specificity is often limited. Although CgA levels correlate with tumor mass, they can be elevated in various other conditions, which reduces their diagnostic utility [12,13,14,15,16]. For patients suspected of having hormonally active GI-NENs, biomarkers such as serotonin, gastrin, norepinephrine, and corticotropin can provide diagnostic clues but often lack precision. These biomarkers are primarily useful for specific tumor types with well-defined clinical syndromes [12,13].

Advances such as the NETest, which uses mRNA profiling, have shown promise in improving diagnostic accuracy, with sensitivity and specificity nearing 99%. However, the NETest has not yet been widely adopted [17,18].

Given these challenges, endoscopic biopsy remains the gold standard for diagnosing NENs in the stomach, duodenum, and colorectum [19]. For small intestinal NENs, video-capsule endoscopy and double-balloon endoscopy (DBE) are effective alternatives, with DBE demonstrating high sensitivity for detecting primary lesions. Computed tomography (CT) and magnetic resonance imaging (MRI) are essential for staging, with MRI being more effective in detecting liver and bone metastases. For functional imaging, 68Ga-DOTATATE PET/CT is preferred due to its superior sensitivity and specificity for somatostatin receptor-expressing tumors [6].

According to the 2019 WHO classification and the 8th edition of the AJCC cancer staging manual, GI-NENs are categorized based on the differentiation into neuroendocrine tumors (NETs) and neuroendocrine carcinomas (NECs). NETs are well differentiated, with further division into grades based on the Ki-67 proliferation index: Grade 1 (Ki-67 < 3%), Grade 2 (Ki-67 between 3% and 20%), and Grade 3 (Ki-67 > 20%). In contrast, NECs are poorly differentiated, with a Ki-67 index typically above 55% [20,21,22].

Tumor resection is the gold standard for the treatment of GI-NENs, as the radical resection of the tumor is the only curative option for patients. Currently, both endoscopic and surgical resections can be performed. Endoscopic resection is recommended only for small (usually less than 1 or 2 cm) well-differentiated NENs in the stomach, duodenum, and rectum. On the other hand, surgical resection is the first line of treatment for small bowel and colon NENs, as well as for more advanced and larger tumors of the stomach, duodenum, and rectum [19]. However, at the time of diagnosis, over 60% of patients have an unresectable tumor or regional or distant metastases; thus, only palliative surgery is possible [23]. Recent studies have shown that aggressive surgical cytoreduction increases overall survival (OS) in patients with NENs, regardless of the tumor stage [24]. Therefore, it is crucial to consider appropriate surgical treatment in each case, taking into account the tumor’s location and stage, as well as the patient’s clinical status [25,26].

In cases of advanced or metastatic GI-NENs, medical treatments including somatostatin analogues (SSAs) and peptide receptor radionuclide therapy (PRRT) are crucial. SSAs are effective in controlling symptoms and slowing disease progression in well-differentiated, somatostatin receptor-positive tumors. Additionally, the NETTER-1 trial has shown that PRRT significantly prolongs progression-free survival [27]. On the other hand, systemic chemotherapy is reserved for more aggressive, poorly differentiated NECs and provides necessary options for managing these challenging cases [19,27].

The main aim of this study is a clinical characterization of GI-NENs and an analysis of the perioperative, short-, and long-term outcomes of the surgical treatment of GI-NENs based on data from the Department of Digestive Tract Surgery of the Medical University of Silesia in Katowice, Poland.

## 2. Materials and Methods

### 2.1. Study Design and Population

The study group consisted of 63 adult patients (32 women, 50.8%; 31 men, 49.2%), aged 30–84 years (mean age 59.7 years, SD 11.84), who underwent surgery for GI-NENs from January 2013 to June 2023 in the Department of Digestive Tract Surgery, Medical University of Silesia in Katowice, Poland.

### 2.2. Study Inclusion/Exclusion Criteria

Our retrospective analysis included patients from the Department of Digestive Tract Surgery at the Medical University of Silesia in Katowice, Poland, who were treated for GI-NENs. Before access, the patients’ data were fully anonymized by the local medical archive. Electronic medical records were individually reviewed. We analyzed only the data of patients diagnosed with GI-NENs by a pathology specialist and who underwent primary surgical tumor resection. Recurrent GI-NENs and mixed adenoneuroendocrine carcinoma (MANEC) diagnosed in histopathology were excluded from the analysis.

### 2.3. Inclusion Criteria for Surgical Treatment

All patients were evaluated by a multidisciplinary team that included surgeons, radiologists, and oncologists, who determined the qualification for a particular type of surgical procedure based on computed tomography (CT) scans. Additionally, 47 patients (74.6%) underwent an endoscopic biopsy with a histopathological diagnosis of GI-NEN prior to surgical treatment. All procedures were performed according to ENETS Guidelines [28,29].

Cytoreduction was defined as the radical resection of the primary tumor in the presence of distant metastasis.

The cohort was divided into three subgroups according to tumor stage: localized (no lymph nodes or distant metastasis), regionally advanced (presence of lymph node metastasis without distant metastasis), and metastatic (presence of distant metastasis).

Overall survival (OS) was measured from the date of the surgical procedure to either the date of death or the date of the last contact. 

### 2.4. Analyzed Data

The study analyzed various parameters related to the patients and their treatment outcomes. These parameters included patients’ general characteristics, such as age, gender, and body mass index (BMI); clinical symptoms; American Society of Anesthesiologists (ASA) score; the type and duration of surgery; surgical margin status (categorized as R0 for no residual tumor, R1 for microscopic residual tumor, or R2 for macroscopic residual tumor); incidence of postoperative complications; reoperations; mortality; duration of hospitalization; rehospitalizations; as well as primary tumor localization, diameter, and released hormones. Additionally, selected pathological parameters were analyzed, including lymphovascular and perineural invasion, histopathological grading, mitotic index, cell proliferation index (Ki-67), lymph node and distant metastases, and follow-up. These parameters were thoroughly examined to gain insights into the clinical characteristics, treatment outcomes, and overall prognosis of patients with GI-NENs.

### 2.5. Statistical Analysis 

The statistical analysis was performed using Statistica^®^ (Tulsa, OK, USA, 2013) software version 13.3 (StatSoft). Absolute values and percentages were used to present qualitative variables, while ranges, means, and standard deviations, or medians with interquartile ranges, were applied for quantitative variables. Predictive factors of postoperative complications were calculated using univariate logistic analysis. Variables identified as significant by univariate analysis were selected for multivariate analysis with logistic regression to identify independent predictors of postoperative complications after the surgical treatment of GI-NENs. Between-group comparisons were performed for two localization categories (foregut and midgut, as there was only one hindgut tumor), in terms of clinical symptoms, released hormones, tumor size, grading, metastases, and recurrence. The analysis was performed using the chi-square test, Fisher’s exact test, or Mann–Whitney U test. Survival analysis was conducted using the Kaplan–Meier estimator, and prognostic factors were analyzed using the Cox proportional hazards regression model. In the survival analysis, patients who were lost to follow-up (3, 4.8%) were treated as censored data. A *p*-value < 0.05 was considered statistically significant.

## 3. Results

### 3.1. General Patient and Tumor Characteristics

In the whole cohort, 42 patients (66.7%) reported clinical symptoms. The most common clinical symptom was abdominal pain, noted in 28 patients (44.4%), followed by carcinoid syndrome in 22 patients (34.9%) and weight loss in 15 patients (23.8%). Most of the tumors (44; 69.8%) were localized in the midgut, with the predominant localization being the ileocecal valve (31; 49.2%).

Eighteen patients (28.6%) underwent systemic therapy before the surgery. The most common treatment was somatostatin analogues, administered to 16 patients (25.4%), followed by FOLFOX chemotherapy (1 patient; 1.6%) and EOX chemotherapy (1 patient; 1.6%). No patients received radiotherapy before surgical treatment.

The most common surgery performed was right hemicolectomy, conducted in 33 patients (52.4%), followed by partial ileal resection in 11 patients (17.5%). Thirty-five patients (55.6%) underwent radical tumor resection, while 28 patients (44.4%) had cytoreduction due to the presence of distant metastases, making radical tumor resection unfeasible. All patients (100%) had surgical margin status classified as R0. Additionally, liver metastases were simultaneously resected in 2 patients (3.2%). Two patients (3.2%) underwent anatomical liver resection: one patient had a bisegmental resection of the 2nd and 3rd liver segments (1.6%), and the other had a resection of the 8th liver segment (1.6%). Intraoperative blood loss was <400 mL in 55 patients (87.3%) and >400 mL in 8 patients (12.7%) (Table 1).

### 3.2. Surgical Outcome

Postoperative complications occurred in 12 patients (19%). The most common postoperative complications following surgical treatment were intra-abdominal abscesses (5 patients; 7.9%), followed by intra-abdominal bleeding (3 patients; 4.8%). Reoperation was necessary for 7 patients (11.1%) due to intra-abdominal bleeding (3 patients; 4.8%), intra-abdominal abscesses (2 patients; 3.2%), and anastomotic leakage (2 patients; 3.2%) (Table 2).

In univariate logistic regression analysis, the occurrence of postoperative complications was associated with patients’ gender (male gender, *p* = 0.02, odds ratio (OR) = 7.5, 95% confidence interval (CI) = 1.4–39.2) and increased surgical procedure duration (*p* = 0.03, OR = 1.5, 95% CI = 1.1–2.2). Conversely, the performance of cytoreduction appeared to be protective against postoperative complications (*p* = 0.04, OR = 0.18, CI = 0.04–0.9). However, this protective effect was not confirmed in multivariate regression analysis (*p* = 0.07, OR = 0.19, CI = 0.03–1.1), indicating that cytoreduction was not an independent predictive factor for postoperative complications. The multivariate logistic regression analysis revealed that patients’ gender (male gender, *p* = 0.04, OR = 6.2, 95% CI = 1.1–35.5) was an independent predictive factor for postoperative complications (Table 3).

One patient (1.6%) required hospitalization in the intensive care unit (ICU) due to pulmonary embolism following a pancreaticoduodenectomy. Two in-hospital deaths (3.2%) were observed after pancreaticoduodenectomy: one due to pulmonary embolism (1.6%) and one due to sepsis following duodeno-jejunal anastomotic leakage (1.6%). There were 3 rehospitalizations (4.8%) for bowel obstruction (1 patient; 1.6%), intra-abdominal abscess (1 patient; 1.6%), and pulmonary embolism (1 patient; 1.6%). Additionally, 7 patients (11.1%) required reoperation, with the most common cause being intra-abdominal abscess (3 patients; 4.8%).

### 3.3. Hormones and Tumor Markers

In the whole group, 46 (73%) patients were hospitalized in the Department of Endocrinology and Neuroendocrine Tumors, Medical University of Silesia, Katowice, Poland before surgical treatment. During their hospitalization, hormonal plasma concentrations and tumor markers were evaluated. The results are shown in Table 4. 

No significant differences were observed between symptomatic and asymptomatic patients in terms of functional tumor and secreting hormones, as well as for most of the hormone and tumor marker levels. The only significant difference observed between symptomatic and asymptomatic patients was in gastrin plasma level (15.3 (0–120, IQR 24.5) pg/mL vs. 10 (0–36.2, IQR 10.2) pg/mL, *p* = 0.02). Nonetheless, none of the patients had a gastrin-secreting GI-NEN; in addition, the median gastrin levels (in symptomatic, asymptomatic, and total patients) were within normal ranges. Therefore, this finding might be coincidental and does not indicate a significant role for elevated gastrin levels in the symptomatology observed in this patient cohort (Table 5).

### 3.4. Tumor Histopathology

The median tumor size was 22 mm (range 1–75 mm, interquartile range [IQR] 27 mm). In pathological staging, 28 tumors (44.4%) were classified as T4. Lymph node metastasis (N1) was found in 44 patients (69.8%), and distant metastasis (M1) was identified in 29 patients (46%). The most common site of distant metastasis was the liver (23 patients; 36.5%). Most tumors (49; 77.8%) were classified as grade 1. Regarding tumor stage, the majority of patients (29; 46.0%) presented with metastatic disease at admission. The median Ki-67 index was 1% (range 0–90%, IQR 2%). Lymphovascular invasion was present in 23 tumors (36.5%), while perineural invasion was observed in only 9 tumors (14.3%) (Table 6).

### 3.5. Comparison between Foregut and Midgut GI-NENs

No statistically significant differences were observed between tumor stage and age or BMI. More patients reported nausea and vomiting in the localized tumor group compared to the regionally advanced and metastatic groups (4; 30.8% vs. 0; 0% vs. 0; 0%, *p* = 0.02). Additionally, more patients reported constipation in the localized tumor group compared to the regionally advanced and metastatic groups (2; 15.4% vs. 0; 0% vs. 0; 0, *p* = 0.04). These results are probably associated with the foregut localization of tumors in patients with nausea and vomiting. There was no statistically significant difference in the occurrence of other clinical symptoms, nor in the overall occurrence of clinical symptoms between groups (Table 7).

More patients presented with foregut tumors in the regionally advanced tumor group compared to the localized and metastatic groups (11; 52.4% vs. 4; 30.8% vs. 3; 10.3%, *p* = 0.01). Additionally, more duodenal tumors were observed in the regionally advanced group compared to the localized and metastatic groups (10; 47.6% vs. 2; 15.4% vs. 2; 6.9%, *p* = 0.002). More metastatic tumors were located in the midgut compared to the localized and regionally advanced tumors (25; 86.2% vs. 9; 69.2% vs. 10; 47.6%, *p* = 0.01), with a higher prevalence of metastatic GI-NENs located at the ileocecal valve compared to localized and regionally advanced GI-NENs (20; 69% vs. 5; 38.5% vs. 6; 28.6%, *p* = 0.01). There were no significant differences between groups in terms of other tumor localizations or ASA scale assessment (Table 7).

More patients were treated with right hemicolectomy in the metastatic NEN group compared to the localized and regionally advanced groups (21; 72.4% vs. 6; 46.2% vs. 6; 28.6%, *p* = 0.008). Additionally, surgical ampullectomy was performed more often in regionally advanced tumors than in localized and metastatic ones (4; 19.1% vs. 0; 0% vs. 0; 0%, *p* = 0.01). These differences could be associated with the fact that midgut tumors, especially those localized at the ileocecal valve, were predominant in the metastatic group (25; 86.2%, with 20; 69.0% at the ileocecal valve), while duodenal tumors dominated in the regionally advanced group (10; 47.6%).

There were no statistically significant differences between the groups in terms of duration of the surgery, blood loss, complications, reoperations, 30-day mortality, duration of hospitalization, and rehospitalizations (Table 8).

**Figure 1:**
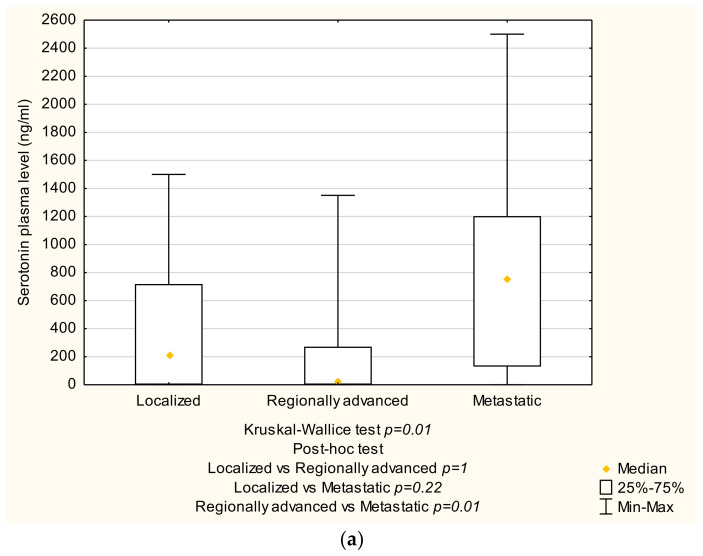

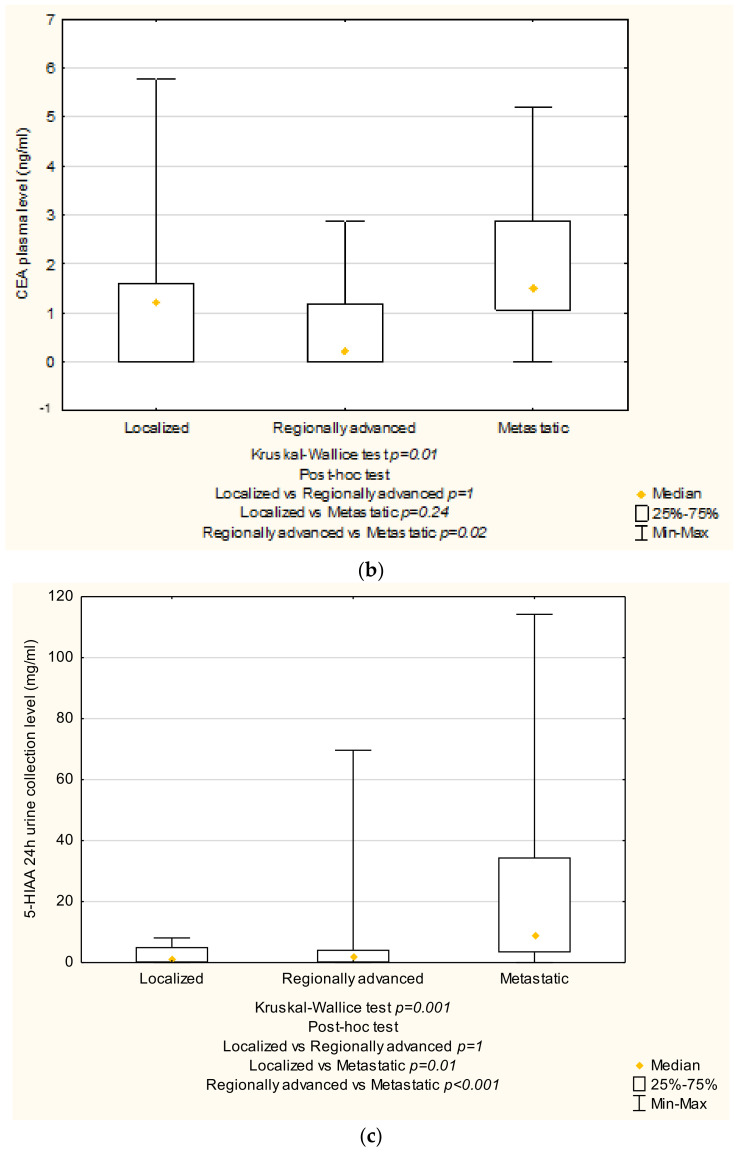
Hormone and tumor marker levels depending on the tumor stage. (**a**) Serotonin (**b**) CEA (**c**) 5-HIAA (Statistica^®^, 13.3, StatSoft). Abbreviations: CEA: Carcino-embryonic antigen, 5HIAA: 5-Hydroxyindoleacetic acid.

There were no significant differences between the groups in terms of tumor size, functional status, released hormones, lymphovascular and perineural invasion, grading, Ki-67 index, and recurrence. However, in patients with metastatic tumors, the serotonin plasma level was higher than in patients with regionally advanced tumors (858.6 (0–2500, IQR 864.5) vs. 167.8 (0–1351, IQR 775.8) mg/mL, *p* = 0.01) (Figure 1a). Additionally, the CEA plasma level was higher among metastatic tumors compared to regionally advanced tumors (1.52 (0.8–5.2, IQR 1.7) vs. 1.1 (0–2.9, IQR 1.9) ng/mL, *p* = 0.02) (Figure 1b). Lastly, in the 24-hour urine collection, the 5-HIAA level was higher in metastatic tumors compared to the localized and regionally advanced tumors (19.3 (1.1–114.2, IQR 32.6) vs. 4.8 (1.1–8.1, IQR 2.7) vs. 4 (0.7–69.6, IQR 5.29), *p* = 0.01 and *p* < 0.001, respectively) (Figure 1c) (Table 9).

### 3.6. Survival Analysis

**Figure 2:**
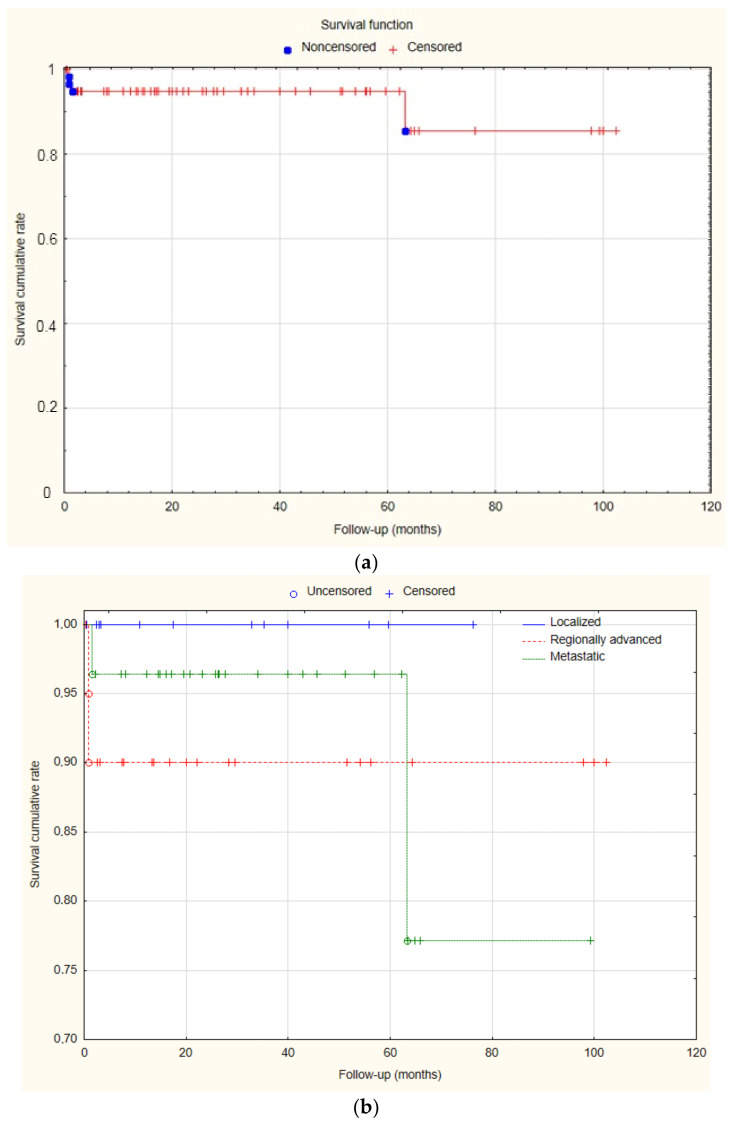
(**a**) Overall survival rate of the series. (**b**) Overall survival depending on tumor stage (Statistica^®^, 13.3, StatSoft).

Median follow-up time was 24.4 (0.37–102, IQR 45.9) months. During the follow-up period, an additional 2 deaths occurred in patients with midgut-located tumors due to cancer cachexia. The two-year OS was 94.9%, while the estimated five-year survival was 94.9%. Additionally, there was no significant difference in two-year OS between localized, regionally advanced and metastatic NENs (100% vs. 90% vs. 96.4%, *p* = 0.46) (Figure 2).

There were no significant differences in overall survival between patients in terms of age, gender, hormonal activity, preoperative medical treatment, tumor localization, tumor grading, distant metastasis, Ki-67 index, tumor diameter, lymphovascular invasion and scope of surgery (Table 10).

During the follow-up, one case of tumor recurrence (1.6%) was observed in a patient with gastric non-secreting neuroendocrine carcinoma (NEC), occurring 22 months after a proximal gastrectomy (Merendino procedure).

## 4. Discussion

GI–NENs are insidious tumors, often presenting with no clinical symptoms for extended periods. Raphael MJ et al. suggest that between 9% and 39% of neuroendocrine tumors in the gastrointestinal tract may be asymptomatic. In our study, 21 patients (33.3%) did not present any symptoms. Additionally, up to 87% of symptomatic patients may exhibit non-specific symptoms [30]. Works in the literature report varying incidences of symptoms for GI-NENs: abdominal pain is observed in 28–79% of patients, followed by bowel obstruction (18–24%), diarrhea (10–32%), weight loss (2–58%), and gastrointestinal bleeding (4–10%) [30,31,32]. In our cohort, abdominal pain was reported by 28 patients (44%), diarrhea by 12 patients (19%), and weight loss by 15 patients (23.8%). The absence of bowel obstruction and gastrointestinal bleeding in our study may be related to the lack of GI-NENs located in the distal colon and the presence of only one rectal tumor. These symptoms are often attributed to alternative diagnoses such as irritable bowel syndrome or dyspepsia [31]. Consequently, the diagnosis of GI-NENs is frequently delayed by 5–7 years, by which time lymph node and/or distant metastases are often already present [2].

The specific symptom of functioning GI-NENs that release serotonin is carcinoid syndrome. This syndrome includes symptoms such as flushing, diarrhea, abdominal cramping, valvular heart disease, telangiectasia, wheezing, pellagra, and paroxysmal tachycardia. Additionally, mesenteric fibrosis can occur in patients with carcinoid syndrome, leading to acute symptoms such as intestinal obstruction and intestinal ischemia, which may require emergency treatment [33]. Carcinoid syndrome is observed in approximately 6–20% of GI-NEN cases, with its incidence being primarily associated with metastatic tumors located in the midgut. This syndrome typically manifests when hormones produced by neuroendocrine tumor cells enter the systemic circulation, usually only after liver metastasis has developed and bypassed hepatic metabolism [31,34].

In our cohort, 22 patients (34.9%) exhibited carcinoid syndrome, with a higher prevalence among those with metastatic tumors (15 patients; 51.7%). This higher incidence in our study may be attributed to the fact that most metastatic tumors originated in the midgut compared to localized and regionally advanced tumors (25 patients; 86.2% vs. 9 patients; 69.2% vs. 10 patients; 47.6%).

Previous studies indicate that between 60% and 93% of gastrointestinal neuroendocrine neoplasms (GI-NENs) are metastatic at the time of diagnosis. The prevalence of lymph node metastases ranges from 14.3% to 34.7%, while distant metastases are reported in 17% to 79.1% of cases. Liver metastases are the most common, occurring in 56% to 84.7% of cases, and are frequently associated with GI-NENs located in the midgut (up to 71%) [29,31,35,36]. In our cohort, 49 patients (77.8%) had lymph node or distant metastases. Specifically, lymph node metastases were present in 44 patients (69.8%), and distant metastases were found in 29 patients (46%). Among the distant metastases, most were located in the liver (23 out of 29). The discrepancy between the incidence of lymph node metastases in the literature (14.3% to 34.7%) and our study (69.8%) may be attributed to the high prevalence of midgut tumors in our cohort (44 patients; 69.8%), where metastases are reported in up to 71% of cases [29,31,35,36].

GI-NENs can be categorized into two groups: functioning and non-functioning. In our study, 30 patients (47.62%) had functioning GI-NENs, while 33 patients (52.38%) had non-functioning ones. Reports in the literature indicate that approximately 65% of GI-NENs are non-functioning [32,35,37]. The higher proportion of functioning GI-NENs observed in our study compared to the literature may be attributed to the predominance of tumors located in the midgut, which accounted for 44 cases (69.8%), as opposed to tumors located in the foregut and hindgut (18 cases, 28.6%, and 1 case, 1.6%, respectively). Functioning GI-NENs are more commonly associated with midgut localization [38]. Additionally, we observed that serotonin plasma levels were higher among patients with metastatic NENs compared to the regionally advanced group ((858.6 (0–2500, IQR 864.5)) vs. 167.8 (0–1351, IQR 775.8) mg/mL, *p* = 0.01), and 5-HIAA levels in 24 h urine collection were higher among patients with metastatic NENs compared to the other groups ((19.3 (1.1–114.2, IQR 32.6) vs. 4.8 (1.1–8.1, IQR 2.7) 4 (0.7–69.6, IQR 5.29), *p* = 0.01 and *p* < 0.001, respectively). Similar findings can be found in the literature, where patients with metastatic NENs had higher serotonin and 5-HIAA levels [39,40]. Other studies stated that patients with more advanced tumors potentially have higher CEA levels [41,42].

There are three main categories for surgical indications in the treatment of GI-NENs: radical or palliative tumor excision, cytoreduction (debulking) of the primary tumor, lymph nodes, or distant metastases, and palliative tumor resection aimed at alleviating symptoms such as obstruction, jaundice, gastrointestinal bleeding, or pain [43,44,45]. In our cohort, radical tumor resection was performed in 35 patients (55.6%), cytoreduction in 28 patients (44.4%), and no patients (0%) received palliative treatment. Rothenstein et al. reported that 62.4% of patients underwent radical tumor resection and 26.6% underwent cytoreduction [37]. The discrepancy in surgical scope between our study and Rothenstein’s may be attributed to the higher proportion of metastatic tumors in our cohort.

The scope and type of surgical procedure for GI-NENs depend significantly on tumor location and staging. Radical resection (R0 resection) of the primary tumor is considered the optimal treatment approach [45]. In our study, all procedures were performed with R0 resection, reflecting an intent to achieve complete tumor removal. For advanced tumors originating from the foregut, partial resection of the affected organ with additional lymphadenectomy may be required. In contrast, advanced neuroendocrine tumors of the midgut generally necessitate a more aggressive approach due to their poorer prognosis compared to other GI-NENs. Notably, the size of midgut tumors often does not correlate with their biological behavior, and metastases can be present even in small primary lesions. Segmental resection with wide lymphadenectomy is typically required for tumors in the jejunum, proximal, and middle ileum, while other midgut tumors may be managed with hemicolectomy and extensive lymphadenectomy. Hindgut tumors are generally treated similarly to foregut tumors [46,47,48].

In our cohort, the majority of patients had GI-NENs located in the midgut (44; 69.8%), with a notable concentration at the ileocecal valve (31; 49.2%). Consequently, the most frequently performed procedure was right hemicolectomy (33; 52.4%). Only 1 patient (1.6%) underwent a local tumor resection with curative intent for a locally advanced 7 mm duodenal tumor.

Regarding the management of distant metastases, particularly liver metastases, several treatment options are currently available. These include somatostatin analogues, mTOR inhibitors such as everolimus, and vascular endothelial growth factor (VEGF) inhibitors like bevacizumab. Additionally, invasive treatments such as metastasectomy, cytoreductive surgery, alcohol ablation, cryoablation, and radiofrequency ablation are also utilized. Somatostatin analogues help alleviate symptoms and exhibit antineoplastic effects, while both everolimus and bevacizumab have been shown to significantly improve progression-free survival. For patients who are contraindicated for metastasis resection, ablation techniques—such as alcohol, cryo-, or radiofrequency ablation—can achieve complete or significant symptom response rates of up to 80% [23]. Despite these available treatments, surgical intervention remains the gold standard for managing GI-NEN metastases, with a notable improvement in five-year survival rates, increasing from 61% to 81% [23]. Research by Tierney et al. has demonstrated that resection of the primary tumor can be a predictive factor for prolonged overall survival [49]. Therefore, whenever feasible, primary tumor resection should be pursued. In our study, 2 out of 29 patients (6.9%) underwent metastasectomy, while 18 out of 29 patients (62.1%) received systemic therapy with somatostatin analogues (16 out of 29; 55.2%) and chemotherapy (1 patient received EOX, and 1 patient received FOLFOX treatment).

Early postoperative complications were observed in 12 (19.1%) patients, with the highest incidence among those with localized tumors (5; 38.5%), compared to regionally advanced (5; 23.8%) and metastatic tumors (2; 6.9%). Postoperative mortality occurred in 2 (3.2%) patients, both with locally advanced neoplasms after pancreaticoduodenectomy. These results align with published data on the treatment of GI-NENs. Works in the literature report postoperative morbidity rates between 5.8% and 7.9%, and mortality rates ranging from 0.5% to 2% [50,51,52]. For localized or regionally advanced tumors, morbidity rates vary between 0% and 39%, while mortality rates range from 0% to 6.9% [52,53]. In our study, the independent predictive factor for postoperative complications was patient gender (male gender, *p* = 0.04, OR = 6.2, 95% CI = 1.1–35.5). Al-Taki et al. also identified gender as a predictive factor for postoperative complications, with female gender associated with a lower risk (*p* < 0.001, OR = 0.7, 95% CI = 0.69–0.71) [38]. This suggests that women may have a lower risk of postoperative complications compared to men. The observed gender differences in preoperative risk factors might be related to males’ tendency to engage less frequently with primary healthcare services, resulting in later-stage surgical interventions. Studies from Canada, the United States, and the United Kingdom have noted that males utilize primary healthcare services less often than females [49,50,51,52,53].

The main limitations of this study include its retrospective design and the fact that it was conducted at a single medical center. The small cohort size may limit the statistical power of the study and affect the robustness of the conclusions drawn. Additionally, the low number of hindgut tumors in the cohort may not provide a comprehensive representation of this tumor type, potentially affecting the generalizability of the findings.

The study also lacked a control group, which restricts the ability to compare outcomes against a non-treatment or alternative treatment group, and thereby limits the assessment of the relative efficacy of the interventions used. Furthermore, selection bias may be present due to the study being conducted at a single institution, which might not reflect the broader population of patients with neuroendocrine tumors. The impact of the COVID-19 pandemic, which resulted in limited access to medical care, could have delayed diagnoses for patients between 2020 and 2022, potentially leading to more advanced tumor stages and influencing the overall outcomes. These factors combined—small sample size, single-center design, lack of a control group, and pandemic-related delays—must be considered when interpreting the results and their applicability to other settings or broader populations.

Nonetheless, this study provides a comprehensive analysis of surgical outcomes and complications associated with GI-NENs in a well-defined cohort. One of its key strengths is the detailed examination of both intraoperative and postoperative complications, offering valuable insights into the surgical management of GI-NENs. The study’s inclusion of a wide range of clinical and pathological parameters enhances the robustness of the findings, allowing for a nuanced understanding of factors influencing surgical outcomes. Additionally, the use of rigorous statistical analyses, including both univariate and multivariate logistic regression, strengthens the validity of the predictive factors identified for postoperative complications. The high rate of R0 resections and the thorough reporting of hormonal and tumor marker levels contribute to a detailed characterization of the disease. Despite the study’s single-center nature, the data collected from a large cohort over an extended period provides a solid foundation for evaluating surgical strategies and outcomes in GI-NENs, thereby advancing the current understanding in this field.

## 5. Conclusions

Symptoms of GI-NENs are mostly non-specific, often leading to late diagnoses when distant or lymph node metastases are already present. Most GI-NENs originate from the midgut, particularly the ileocecal valve. Metastatic GI-NENs are associated with midgut localization, and these tumors are often hormonally active, additionally exhibiting higher levels of serotonin, 5-HIAA, and CEA compared to less advanced NENs. Postoperative morbidity and mortality in patients undergoing surgery for GI-NENs are generally low. What is more, the male gender can be a predictive factor for postoperative complications after surgical treatment of GI-NENs The overall prognosis for GI-NENs is favorable, with high overall survival rates.

## Figures and Tables

**Table 1:** General characteristics of patients undergoing surgery due to gastrointestinal neuroendocrine neoplasms.

Variable	n (%); Mean/Median (Range, SD/IQR)
Age	59.7 (30–84, SD 11.84)
Gender	
Female	32 (50.8%)
Male	31 (49.2%)
Presence of comorbidities (arterial hypertension, diabetes mellitus, history of myocardial infarction/stroke, COPD, asthma and others)	47 (74.6%)
MEN I, II (yes)	0
Cigarette smoking (yes)	13 (20.6%)
Previous abdominal surgeries (yes)	28 (44.4%)
Clinical symptoms (yes)	42 (66.7%)
Abdominal pain	28 (44.4%)
Carcinoid syndrome	22 (34.9%)
Weight loss	15 (23.8%)
Diarrhea	12 (19%)
Nausea, vomiting	4 (6.3%)
Bloating	4 (6.3%)
Jaundice	2 (3.2%)
Constipation	2 (3.2%)
Fever	2 (3.2%)
Preoperative medical treatment (yes)	18 (28.6%)
Somatostatin	16 (25.4%)
FOLFOX	1 (1.6%)
EOX	1 (1.6%)
ASA Score	
I	3 (4.8%)
II	33 (52.4%)
III	27 (42.9%)
Tumor location	
Foregut	18 (28.6%)
Stomach	4 (6.3%)
Duodenum	14 (22.2%)
Midgut	44 (69.8%)
Ileocecal valve	31 (49.2%)
Ileum	12 (19%)
Appendix	1 (1.6%)
Hindgut	1 (1.6%)
Rectum	1 (1.6%)
Type of surgery	
Right hemicolectomy	33 (52.4%)
Partial ileal resection	11 (17.5%)
Pancreaticoduodenectomy	6 (9.5%)
Traverso procedure	5 (7.9%)
Whipple procedure	1 (1.6%)
Surgical ampullectomy	4 (6.3%)
Partial gastrectomy	3 (4.7%)
Proximal gastrectomy—Merendino procedure	1 (1.6%)
Distal gastrectomy—Roux-Y reconstruction	1 (1.6%)
Subtotal gastrectomy—Billroth II with Braun reconstruction	1 (1.6%)
Distal duodenectomy (resection of 3rd and 4th part of duodenum)	3 (4.8%)
Total gastrectomy with D2 lymphadenectomy—Roux-Y reconstruction	1 (1.6%)
Local resection of duodenal tumor	1 (1.6%)
Local resection of the rectal tumor	1 (1.6%)
Scope of surgery	
Radical tumor resection	35 (55.6%)
Cytoreduction	28 (44.4%)
Surgical margin status	
R0	64 (100%)
R1	0 (0%)
R2	0 (0%)
Treatment of liver metastasis (yes)	2 (3.2%)
The anatomical liver resection	2 (3.2%)
Intraoperative blood loss	
<400 mL	55 (87.3%)
>400 mL	8 (12.7%)
Surgical procedure duration (minutes)	228 (60–715, IQR 99)
Total duration of hospitalization (days)	10 (6–75, IQR 4)

Abbreviations: SD—standard deviation, IQR—interquartile range, BMI—body mass index, COPD—chronic obstructive pulmonary disease, MEN—multiple endocrine neoplasia, ASA—American Society of Anesthesiologists.

**Table 2:** Postoperative complications.

Complications (n = 12, 19%)	Surgical Treatment
Intra-abdominal abscess (5; 7.9%)	Right hemicolectomy
	Pancreaticoduodenectomy
Intra-abdominal bleeding (3, 4.8%)	Right hemicolectomy
	Partial ileal resection
Anastomotic leakage (3; 4.8%)	
Duodeno-jejunal anastomosis (2; 3.2%)	Pancreaticoduodenectomy
Ileo-transverse anastomosis (1; 1.6%)	Right hemicolectomy
Pulmonary embolism (1; 1.6%)	Pancreaticoduodenectomy

**Table 3:** Predictive factors of postoperative complications after surgical treatment of GI-NENs in univariate and multivariate logistic regression analysis.

	Univariate Analysis				Multivariate Analysis		
Variable	n	OR	95% CI	*p*	OR	95% CI	*p*
Preoperative							
Age		1.1	1–1.1	0.14			
Gender				0.02			
Male	31	7.5	1.4–39.2		6.2	1.1–35.5	0.04
Female	32	1			1		
BMI		1	0.9–1.1	0.87			
Presence of comorbidities (yes)	47	1.94	0.4–10.4	0.43			
Cigarette smoking (yes)	13	0.98	0.9–1.1	0.63			
Previous abdominal surgeries (yes)	28	1.3	0.4–4.6	0.71			
Clinical symptoms (yes)	42	1	0.3–4	0.96			
Preoperative medical treatment (yes)	18	1.3	0.3–5.1	0.72			
ASA score				0.09			
3	36	3.3	0.8–12.7				
≤2	27	1					
Tumor origin				0.72			
Foregut	18	0.8	0.2–3.1				
Midgut	44	1					
Intra-operative							
Intent of surgery				0.04			0.07
Radical tumor resection	35	1	6		1		
Cytoreduction	28	0.18	0.04–0.9		0.19	0.03–1.1	
Blood loss				0.67			
>400 mL	8	1.5	0.24–8.7				
<400 mL	55	1					
Duration of surgical procedure		1.5	1.1–2.2	0.03	1.4	0.9–2.1	0.12

Abbreviations: OR—odds ratio, CI—confidence interval, BMI—body mass index, ASA—American Society of Anesthesiologists.

**Table 4:** Hormone and tumor marker results for patients with GI-NENs.

Variable	n (%); Mean/Median (Range, SD/IQR)
Functioning tumor (yes)	30 (47.6%)
Serotonin secreting	28 (44.4%)
Somatostatin secreting	1 (1.6%)
Norepinephrine secreting	1 (1.6%)
Chromogranin A (plasma) level	9.7 (0–2139, IQR 107.9) ng/mL
Serotonin (plasma) level	718 (0–2500, IQR 1007.2) ng/mL
Gastrin (plasma) level	12 (0–120, IQR 31.7) pg/mL
CA19.9 (plasma) level	5.1 (0–32.57, IQR 6.4) U/mL
CEA (plasma) level	1.5 (0–5.78, IQR 1.6) ng/mL
5-HIAA (24 h urine collection) level	7.5 (0.7–114.2, IQR 22.5) mg/mL

Abbreviations: CA19.9—Carbohydrate antigen 19.9, CEA—Carcinoembryonic antigen, 5-HIAA—5-Hydroxy indoleacetic acid, SD—standard deviation, IQR—interquartile range.

**Table 5:** Occurrence of symptoms depending on the hormone and tumor marker levels in patients with GI-NENs.

Variable	Symptomatic (42; 66.7%)	Asymptomatic (21; 33.3%)	Total (63)	*p*
Functional tumor (yes)	22 (52.4%)	8 (38.1%)	30 (47.62%)	0.28
Serotonin secreting	20 (46.6%)	8 (38.1%)	28 (44.4%)	0.47
Somatostatin secreting	1 (2.4%)	0 (0%)	1 (1.59%)	1
Norepinephrine secreting	1 (2.4%)	0 (0%)	1 (1.59%)	1
Chromogranin A (plasma) level	84.14 (0–2139, IQR 106.04) ng/mL	50.14 (20.85–232.71, IQR 50.59) ng/mL	9.7 (0–2139, IQR 107.9) ng/mL	0.09
Serotonin (plasma) level	718.82 (0–2500, IQR 1003.04) ng/mL	692.96 (10.29–2102, IQR 1017.17) ng/mL	718 (0–2500, IQR 1007.2) ng/mL	0.59
Gastrin (plasma) level	15.3 (0–120, IQR 24.5) pg/mL	10 (0–36.2, IQR 10.2) pg/mL	12 (0–120, IQR 31.7) pg/mL	0.02
CA19.9 (plasma) level	4.59 (0–32.45, IQR 6.63) U/mL	5.97 (0–32.57, IQR 4.41) U/mL	5.1 (0–32.57, IQR 6.4) U/mL	0.44
CEA (plasma) level	1.49 (0–5.78, IQR 1.65) ng/mL	1.85 (0.93–2.9, IQR 0.89) ng/mL	1.5 (0–5.78, IQR 1.6) ng/mL	0.81
5-HIAA (24 h urine collection) level	5.57 (0.7–114.2, IQR 20.11) mg/mL	8.09 (1.69–37.09, IQR 22.53) mg/mL	7.5 (0.7–114.2, IQR 22.5) mg/mL	0.87

Abbreviations: CA19.9—Carbohydrate antigen 19.9, CEA—Carcinoembryonic antigen, 5-HIAA—5-Hydroxy indoleacetic acid, SD—standard deviation, IQR—interquartile range.

**Table 6:** GI-NEN histopathology.

Variable	n (%); Mean/Median (Range, SD/IQR)
Tumor size (mm)	22 (1–75) IQR 27 mm
Pathological staging	
T	
T1	9 (14.3%)
T2	13 (20.6%)
T3	13 (20.6%)
T4	28 (44.4%)
N	
0	19 (30.2%)
1	44 (69.8%)
M	
0	34 (54%)
1	29 (46%)
Location of distant metastasis	
Liver	23 (36.5%)
Peritoneum	5 (7.9%)
Bones	1 (1.6%)
Kidneys	1 (1.6%)
Tumor stage	
Localized	13 (20.6%)
Regionally advanced	21 (33.3%)
Metastatic	29 (46.0%)
Grading	
G1	49 (77.8%)
G2	5 (7.9%)
G3	9 (14.3%)
Type of neuroendocrine neoplasm	
Neuroendocrine tumor	54 (85.7%)
Neuroendocrine carcinoma	9 (14.3%)
Ki-67% index	1 (0–90 IQR 2) %
Lymphovascular invasion (yes)	23 (36.5%)
Perineural invasion (yes)	9 (14.3%)

Abbreviations: SD—standard deviation, IQR—interquartile range.

**Table 7:** Patients’ characteristics by stage of GI-NEN.

Tumor Stage	Localized (13; 20.6%)	Regionally Advanced (21; 33.3%)	Metastatic (29; 46.0%)	Total (63)	*p* (df = 1)
Age	62.92 (40–84, SD 14.47)	56 (30–74, SD 12.47)	60.62 (40–81, SD 9.13)	59.7 (30–84, SD 11.84)	0.19
Gender					0.59
Female	5 (38.5%)	11 (52.4%)	16 (55.2%)	32 (50.8%)	
Male	8 (61.5%)	10 (47.6%)	13 (44.8%)	31 (49.2%)	
BMI	28 (22.7–34.2, IQR 4.5)	25.8 (20.6–34.5, IQR 5.7)	25.7 (18.4–43.7, IQR 5.1)	25.8 (18.4–43.8, IQR 5.81)	0.94
Comorbidities (yes)	10 (76.9%)	17 (81.0%)	20 (69.0%)	47 (74.6%)	0.61
Clinical symptoms (yes)	11 (84.6%)	13 (61.9%)	18 (62.1%)	42 (66.7%)	0.3
Abdominal pain	9 (69.2%)	10 (47.6%)	9 (31.0%)	28 (44.4%)	0.07
Carcinoid syndrome	2 (15.4%)	5 (23.8%)	15 (51.7%)	22 (34.9%)	0.03
Weight loss	4 (30.8%)	3 (14.3%)	8 (27.6%)	15 (23.8%)	0.44
Diarrhea	2 (15.4%)	4 (19.1%)	5 (17.2%)	12 (19%)	0.96
Nausea, vomiting	4 (30.8%)	0 (0%)	0 (0%)	4 (6.3%)	0.02
Bloating	1 (7.7%)	2 (9.5%)	1 (3.5%)	4 (6.3%)	0.66
Jaundice	0 (0%)	1 (4.8%)	1 (3.5%)	2 (3.2%)	0.73
Constipation	2 (15.4%)	0 (0%)	0 (0%)	2 (3.2%)	0.04
Fever	1 (7.7%)	1 (4.8%)	0 (0%)	2 (3.2%)	0.37
Tumor localization					
Foregut	4 (30.8%)	11 (52.4%)	3 (10.3%)	18 (28.6%)	0.01
Stomach	2 (15.4%)	1 (4.8%)	1 (3.5%)	4 (6.3%)	0.32
Duodenum	2 (15.4%)	10 (47.6%)	2 (6.9%)	14 (22.2%)	0.002
Midgut	9 (69.2%)	10 (47.6%)	25 (86.2%)	44 (69.8%)	0.01
Ileocecal valve	5 (38.5%)	6 (28.6%)	20 (69.0%)	31 (49.2%)	0.01
Ileum	3 (23.1%)	4 (19.1%)	5 (17.4%)	12 (19%)	0.9
Appendix	1 (7.7%)	0 (0%)	0 (0%)	1 (1.6%)	0.14
Hindgut	0 (0%)	0 (0%)	1 (3.5%)	1 (1.6%)	0.6
Rectum	0 (0%)	0 (0%)	1 (3.5%)	1 (1.6%)	0.6
ASA Score					0.05
I	1 (7.7%)	2 (9.5%)	0 (0%)	3 (4.8%)	
II	3 (23.1%)	14 (66.7%)	16 (55.2%)	33 (52.4%)	
III	9 (69.2%)	5 (23.8%)	13 (44.8%)	27 (42.9%)	

Abbreviations: SD—standard deviation, IQR—interquartile range, BMI—body mass index, ASA—American Society of Anesthesiologists.

**Table 8:** Surgery characteristics by stage of GI-NEN.

Tumor Stage	Localized (13; 20.6%)	Regionally Advanced (21; 33.3%)	Metastatic (29; 46.0%)	Total (63)	*p* (df = 1)
Type of the surgery					
Right hemicolectomy	6 (46.2%)	6 (28.6%)	21 (72.4%)	33 (52.4%)	0.008
Partial ileal resection	3 (23.1%)	4 (19.1%)	4 (13.8%)	11 (17.5%)	0.74
Pancreaticoduodenectomy	0 (0%)	5 (23.8%)	1 (3.5%)	6 (9.5%)	0.02
Traverso procedure	0 (0%)	5 (23.8%)	0 (0%)	5 (7.9%)	N/A
Whipple procedure	0 (0%)	0 (0%)	1 (3.5%)	1 (1.6%)	
Surgical ampullectomy	0 (0%)	4 (19.1%)	0 (0%)	4 (6.3%)	0.01
Partial gastrectomy	2 (15.4%)	1 (4.8%)	0 (0%)	3 (4.7%)	0.3
Proximal gastrectomy—Merendino procedure	1 (7.7%)	0 (0%)	0 (0%)	1 (1.6%)	N/A
Distal gastrectomy—Roux-Y reconstruction	1 (7.7%)	0 (0%)	0 (0%)	1 (1.6%)	
Subtotal gastrectomy—Billroth II with Braun reconstruction	0 (0%)	1 (4.8%)	0 (0%)	1 (1.6%)	
Distal duodenectomy (resection of 3rd and 4th part of duodenum)	1 (7.7%)	1 (4.8%)	1 (3.5%)	3 (4.8%)	0.84
Total gastrectomy with D2 lymphadenectomy—Roux-Y reconstruction	0 (0%)	0 (0%)	1 (3.5%)	1 (1.6%)	0.55
Local resection of duodenal tumor	1 (7.7%)	0 (0%)	0 (0%)	1 (1.6%)	0.14
Local resection of rectal tumor	0 (0%)	0 (0%)	1 (3.5%)	1 (1.6%)	0.55
Duration of surgery (minutes)	210 (90–400, IQR 50)	245 (130–715, IQR 139)	240 (60–410, IQR 70)	228 (60–715, IQR 99)	0.36
Blood loss					0.33
<400 mL	10 (76.9%)	18 (85.7%)	27 (93.1%)	55 (87.3%)	
>400 mL	3 (23.1%)	3 (14.3%)	2 (6.9%)	8 (12.7%)	
Complications (yes)	5 (38.5%)	5 (23.8%)	2 (6.9%)	12 (19%)	0.06
Intra-abdominal abscess	2 (15.4%)	3 (14.3%)	0 (0%)	5 (7.9%)	0.09
Intra-abdominal bleeding	1 (7.7%)	1 (4.8%)	1 (3.5%)	3 (4.8%)	0.84
Anastomotic leakage	1 (7.7%)	2 (9.5%)	0 (0%)	3 (4.8%)	0.25
Duodeno-jejunal anastomosis	0 (0%)	2 (9.5%)	0 (0%)	2 (3.2%)	N/A
Ileo-transverse anastomosis	1 (7.7%)	0 (0%)	0 (0%)	1 (2.3%)	N/A
Pulmonary embolism	0 (0%)	0 (0%)	1 (3.5%)	1 (1.6%)	1
Reoperations	2 (15.4%)	3 (14.3%)	2 (6.9%)	7 (11.1%)	0.61
30-day mortality	0 (0%)	2 (9.5%)	0 (0%)	2 (3.2%)	0.8
Duration of hospitalization (days)	10 (7–21, IQR 5.5)	10 (1–75, IQR 7)	9 (6–33, IQR 2)	10 (6–75, IQR 4)	0.53
Rehospitalization (yes)	2 (15.4%)	1 (4.8%)	0 (0%)	3 (4.8%)	0.09

Abbreviations: IQR—interquartile range, N/A—not applicable.

**Table 9:** Tumor characteristics by stage of GI-NEN.

Tumor Stage	Localized (13; 20.6%)	Regionally Advanced (21; 33.3%)	Metastatic (29; 46.0%)	Total (63)	*p* (df = 1)
Tumor size (mm)	24.5 (6–70, IQR 37.5)	19 (1–75, IQR 23)	27 (2–70, IQR 23)	22.5 (1–75, IQR 25)	0.62
Functioning tumor	6 (46.2%)	7 (33.3%)	17 (58.6%)	30 (47.6%)	0.21
Serotonin	6 (46.2%)	6 (28.6%)	16 (55.2%)	28 (44.4%)	0.55
Somatostatin	0 (0%)	1 (4.8%)	0 (0%)	1 (1.59%)	0.36
Norepinephrine	0 (0%)	0 (0%)	1 (3.5%)	1 (1.59%)	0.6
Hormones and tumor markers levels					
Chromogranin A (plasma) (ng/mL)	56.1 (19.1–700, IQR 109.7)	46.5 (0–2139, IQR 249.2)	77.6 (17.1–1080.2, IQR 88.96)	9.7 (0–2139, IQR 107.9)	0.11
Serotonin (plasma) (ng/mL)	640.6 (9.1–1500, IQR 804.9)	167.8 (0–1351, IQR 775.8)	858.6 (0–2500, IQR 864.5)	718 (0–2500, IQR 1007.2)	0.01
Gastrin (plasma) (ng/mL)	42.5 (0–120, IQR 49.7)	10 (0–67.1, IQR 20.1)	12 (0–55, IQR 14)	12 (0–120, IQR 31.7)	0.56
CA19.9 (plasma) (U/mL)	4.1 (0–19.1, IQR 5.5)	4.6 (0–32.5, IQR 9.9)	6.0 (0–32.6, IQR 6.8)	5.1 (0–32.57, IQR 6.4)	0.08
CEA (plasma) (ng/mL)	1.6 (0–5.8, IQR 0.6)	1.1 (0–2.9, IQR 1.9)	1.52 (0.8–5.2, IQR 1.7)	1.5 (0–5.78, IQR 1.6)	0.01
5-HIAA (24 h Urine collection) (mg/mL)	4.8 (1.1–8.1, IQR 2.7)	4 (0.7–69.6, IQR 5.29)	19.3 (1.1–114.2, IQR 32.6)	7.5 (0.7–114.2, IQR 22.5)	0.001
Lymphovascular invasion (yes)	2 (15.4%)	8 (38.1%)	13 (44.8%)	23 (36.5%)	0.69
Perineural invasion (yes)	1 (7.7%)	3 (14.3%)	5 (17.2%)	9 (14.3%)	0.71
Grading					0.51
G1	11 (84.6%)	16 (76.2%)	22 (75.86%)	49 (77.8%)	
G2	0 (0%)	1 (4.8%)	4 (13.8%)	5 (7.9%)	
G3	2 (15.4%)	4 (19.1%)	3 (10.3%)	9 (14.3%)	
Ki-67 index (%)	1 (0–90, IQR 0)	1 (1–70, IQR 2)	1 (0–80, IQR 2)	1 (1–90, IQR 2)	0.18
Recurrence (yes)	0 (0%)	0 (0%)	1 (3.5%)	1 (1.6%)	0.29

Footnote: Between-group differences were tested for foregut and midgut tumors. Abbreviations: SD—standard deviation, IQR—interquartile range, CA19.9—Carbohydrate antigen 19.9, CEA—Carcinoembryonic antigen, 5-HIAA—5-Hydroxy indoleacetic acid, N/A—not applicable.

**Table 10:** Univariate analysis of factors with overall survival using Cox proportional hazards regression model.

	Univariate Analysis			
Variable	Survival Time (months)	HR	95% CI	*p*
Age		1.03	0.95–1.12	0.47
Gender				0.95
Male	28.9 (0.5–102.4, IQR 48.1)	1.05	0.1–7.5	
Female	17.3 (0.4–97.8, IQR 41.9)	1		
Functioning tumor				0.23
Yes	30.3 (2.4–102.4, IQR 45)	0.2	0.03–2.5	
No	16.4 (0.4–76.3, IQR 44)	1		
Preoperative medical treatment				0.58
Yes	34.8 (2.4–99.9, IQR 42.6)	0.5	0.05–5.3	
No	17.8 (0.4–102.4, IQR 41.8)	1		
Localization				0.26
Midgut	27 (0.5–102.4, IQR 39.8)	0.3	0.04–2.3	
Foregut	15.5 (0.4–64.9, IQR 50.9)	1		
Grading				
G1	24.2 (0.4–102.4, IQR 45)	1		
G2	23.1 (1.5–64.9, IQR 44.8)	3.4	0.3–38.7	0.4
G3	25.7 (0.9–76.3, IQR 23.9)	2.8	0.3–31.2	0.4
Type of neuroendocrine neoplasm				0.45.
Neuroendocrine carcinoma	25.7 (0.9–76.3, IQR 23.9)	2.4	0.25–23.3	
Neuroendocrine tumor	23.1 (0.4–103.4, IQR 45.9)	1		
Distant metastasis				0.96
Yes	26.4 (1.5–99.3, IQR 38.60)	1.1	0.14–7.5	
No	18.7 (0.4–102.4, IQR 50.9)	1		
Localization				
Ki-67 index		1.01	0.98–1.04	0.47
Tumor diameter	15 IQR 32	0.96	0.9–1.03	0.27
Lymphovascular invasion				0.47
Yes	26.4 (0.4–97.8, IQR 38.2)	2.1	0.3–15.5	
No	22 (0.5–102.4, IQR 48.6)	1		
Scope of surgery				0.9
Radical tumor resection	18.7 (0.4–102.4, IQR 51)	1.1	0.4–2.9	
Cytoreduction	26.4 (1.5–99.3, IQR 42)	1		

Abbreviations: HR—hazard ratio, CI—confidence interval.

## Data Availability

The raw data supporting the conclusions of this article will be made available by the authors on request.
